# Evaluation of Blood Pressure Variability With Clevidipine Infusion in Patients With Acute Cerebrovascular Disease

**DOI:** 10.7759/cureus.108238

**Published:** 2026-05-04

**Authors:** Caitlin S Brown, Tuba Chaudhry, Kristin Cole, Alejandro Rabinstein

**Affiliations:** 1 Pharmacy, Mayo Clinic, Rochester, USA; 2 Neurology, Mayo Clinic, Rochester, USA; 3 Quantitative Health Sciences, Mayo Clinic, Rochester, USA

**Keywords:** blood pressure, cerebrovascular disease, clevidipine, intracranial hemorrhages, neurology and critical care

## Abstract

Background: Blood pressure variability (BPV) has been associated with worse outcomes in patients with acute cerebrovascular diseases, and acute hypertension is routinely treated with intravenous antihypertensives.

Objective: The goal of this study was to determine if clevidipine infusions can safely and effectively reach blood pressure targets and maintain blood pressure control without undesirable variability.

Methods: This was a prospective, single-arm, observational study. We enrolled a convenience sample of adult patients hospitalized for acute ischemic stroke (AIS), intracerebral hemorrhage (ICH), subarachnoid hemorrhage (SAH), or posterior reversible encephalopathy syndrome (PRES) who required clevidipine for blood pressure control within 24 hours of symptom onset. The primary efficacy outcome was systolic BPV during the first hour. The primary safety outcomes were acute neurological decline and acute kidney injury (AKI). Secondary outcomes were BPV within 24 hours, time to blood pressure goal, and the association between BPV and early neurologic decline and AKI.

Results: Thirty patients were enrolled with a median age of 64 (58, 77), and most were White (83.3%) and females (57%). The most common primary diagnosis was ICH (63%). The median (IQR) first systolic blood pressure (SBP) reading was 170 (153, 193) mmHg. The median (interquartile range (IQR)) time from clevidipine initiation to blood pressure goal was 20 (10, 28) minutes. The mean systolic BPV in the first hour, as measured by standard deviation (SD), was 17.2 (9.3). At 24 hours, the mean systolic BPV by SD was 16.4 (5.6). Six patients (20%) experienced AKI, and 11 (37%) experienced a neurologic decline.

Conclusion: This study describes BPV observations with clevidipine in cerebrovascular disease. This study is limited by the small sample size and observational design. Comparative studies evaluating the effects of different antihypertensive strategies on BPV are needed.

## Introduction

Blood pressure variability (BPV) is the variation in blood pressure over a given time and has been associated with worse outcomes in patients with acute cerebrovascular diseases, including acute ischemic stroke (AIS), intracerebral hemorrhage (ICH), and nontraumatic subarachnoid hemorrhage (SAH) [1-10]. In patients with injured brains, cerebral autoregulation is significantly impaired, particularly in the acute period. Acute hypertension in neurologic emergencies is routinely treated with intravenous bolus doses of antihypertensives followed by continuous infusions if targets are not reached [10]. There is some evidence, albeit limited, that continuous infusion antihypertensives may be associated with less BPV when compared to intravenous bolus doses [11]. The goal of this study was to determine if clevidipine infusion can safely and effectively reach blood pressure targets without delays and maintain blood pressure control without undesirable variability in patients with neurologic emergencies.

## Materials and methods

This was a prospective, single-arm, observational study. We enrolled a convenience sample of adult patients hospitalized for AIS, ICH, SAH, or posterior reversible encephalopathy syndrome (PRES) who required clevidipine for blood pressure control within 24 hours of symptom onset. Patients receiving clevidipine for acute cerebrovascular disease were initially identified by neurocritical care physicians, nurse practitioners, neurocritical care fellows, and pharmacists. Once patients were identified by the care team to be on the clevidipine study, the investigators then screened participants for inclusion. Patients were included in the analysis if they received clevidipine for a minimum of six hours within the first 24 hours of intensive care unit (ICU) admission. Patients who were pregnant, already had their blood pressure controlled with another agent, or did not have authorization for research in the state of Minnesota were excluded. An a priori sample of 30 patients was established, and patients were enrolled between 10/31/2021 and 8/23/2022. Clevidipine was started per our institutional protocol at 1 mg/hour and titrated in 2 mg/hour increments every 5-15 minutes to a maximum of 21 mg/hour. Blood pressure is monitored every five minutes with continuous blood pressure or noninvasive blood pressure. Clevidipine must be administered in the intensive care unit (ICU) at our hospital. This study was approved by the Institutional Review Board (IRB# 21-007375). The primary efficacy outcome was to measure systolic BPV during the first hour by the definitions here: 

Mean: ${x} = \frac{\sum_{i=1}^{n} x_i}{n}$

Standard deviation (SD): $SD = \sqrt{\frac{\sum_{i=1}^{n} (x_i - \bar{x})^2}{n-1}}$

Coefficient of variation (CV): $CV = 100 \times \frac{SD}{\bar{x}}$

Variation independent of mean (VIM): $VIM = k \times \frac{SD}{\bar{x}^m}$

Residual standard deviation (RSD): $RSD = \sqrt{\frac{\sum_{i=1}^{n} (x_i - \hat{x}_i)^2}{n - 2}}, \text{ where } \hat{x}_i \text{ are fitted values from a linear regression of BP against time.}$

Average real variability (ARV): $ARV = \frac{1}{n - 1} \sum_{i=1}^{n - 1} |x_{i+1} - x_i|$

Successive variation (SV): $SV = \sqrt{\frac{1}{n - 1} \sum_{i=1}^{n - 1} (x_{i+1} - x_i)^2}$

The primary safety outcomes were to determine rates of acute neurological decline and acute kidney injury (AKI). The secondary outcomes were to measure BPV within 24 hours, determine time to blood pressure target, and evaluate if there is an association between BPV and neurologic decline, AKI, and time to goal blood pressure from initiation of clevidipine. The blood pressure target was identified via the medication order in the electronic medical record.

Baseline demographics, blood pressures, hypotension (defined as systolic blood pressure (SBP) < 90mmHg), type of blood pressure monitoring (arterial versus cuff pressure), clevidipine dosing, AKI, neurologic decline, and discharge three-month Modified Rankin Scale (mRS) scores were collected from the electronic health record. AKI was defined by the KDIGO guidelines, and early neurologic decline was defined as the National Institute of Health Stroke Scale (NIHSS) increase of ≥4 or Glasgow Coma Scale (GCS) sum score drop of ≥2 in the first 24 hours [12].

Data were summarized using means and standard deviations or medians and interquartile ranges for continuous data and frequencies and percentages for categorical data. The association between BPV (within one hour and 24 hours) and the outcomes of early neurologic decline and AKI injury was assessed using Wilcoxon rank sum tests. Linear regression was used to assess the association between BPV (within one hour and 24 hours) and time to achieve the goal blood pressure. All analyses were performed using SAS version 9.4 software (SAS Institute, Inc.; Cary, NC).

## Results

Thirty patients were enrolled with a median age of 64 (58, 77), and most were white (83.3%, n = 25) and female (57%, n = 17) (Table 1). The most common primary diagnosis was ICH (63%, n = 19), followed by AIS (20%, n = 6). At one hour, six patients had blood pressure measurements available via an arterial line, and at 24 hours, 13 patients had blood pressure measurements available via an arterial line. BPV at one hour is shown in Table 2, and BPV at 24 hours is shown in Table 3. Given that ICH accounted for 63% (n = 19) of patients, ARV was calculated for the subgroup of ICH patients (Table 4). The median time (IQR) from clevidipine initiation to blood pressure target was 20 minutes (10, 28).

**Table 1:** Baseline demographics SBP: systolic blood pressure; DBP: diastolic blood pressure; IQR: interquertile range Data is presented as N (%) or median (IQR)

	N = 30
Age (median (IQR))	64 (58, 77)
Race/ethnicity, n (%)	-
White	25 (83.3)
Black	2 (6.7)
Hispanic	2 (6.7)
Other	1 (3.3)
Sex (male), n (%)	13 (43.3)
History of hypertension, n (%)	27 (90)
Primary diagnosis, n (%)	-
Intracranial hemorrhage (ICH)	19 (63.3)
Acute ischemic stroke (AIS)	6 (20)
Aneurysmal subarachnoid hemorrhage (aSAH)	2 (6.7)
Posterior reversible encephalopathy syndrome (PRES)	3 (10)
Diabetes	16 (53.3)
Baseline Modified Rankin Scale (mRS) (median (IQR))	0 (0,1)
Baseline Glascow Coma Scale (GCS) (median (IQR))	12 (7, 14)
First SBP (median (IQR))	170 (153, 193)
Goal blood pressure, n (%)	-
SBP < 140	7 (23.3)
SBP < 160	14 (46.7)
SBP < 180	7 (23.3)
SBP < 200	1 (3.3)
DBP < 105	1 (3.3)
SBP (<90), n (%)	6 (20)
Duration of clevidipine (hh:mm) (median (IQR))	22:21 (16:34, 37:47)
Median dose of clevidipine, mg/hour, (median (IQR))	4 (2,8)
Average hourly dose (adjusted for duration on each dose), mg/hour, (median (IQR))	4 (2.4, 8.5)

**Table 2:** Blood pressure variability within one hour of clevidipine SBP: systolic blood pressure; DBP: diastolic blood pressure; SD: standard deviation; IQR: interquartile range; ARV: average real variability; SV: successive variation; CV: coefficient of variation Data is presented as mean (SD) or median (IQR)

	Arterial measures within 1 hour of clevidipine start			All Measures within 1 hour of clevidipine start		
	SBP (N = 6)	DBP (N = 6)	MAP (N = 6)	SBP (N = 30)	DBP (N = 30)	MAP (N = 30)
Mean						
Mean (SD)	168.1 (53.0)	72.4 (15.3)	103.2 (10.3)	158.2 (24.6)	86.1 (15.9)	108.4 (14.1)
Median (IQR)	173.0 (164.7, 201.7)	74.7 (68.1, 76.5)	104.3 (96.3, 110.0)	161.5 (138.8, 173.3)	87.5 (72.4, 95.8)	106.4 (95.7, 121.4)
Range	(81.9-219.5)	(50.2-92.5)	(89.9-115.5)	(90.2-206.6)	(50.2-114.0)	(88.6-132.2)
SD						
Mean (SD)	16.2 (5.6)	10.2 (6.7)	26.9 (28.5)	17.2 (9.3)	13.0 (7.9)	14.7 (12.8)
Median (IQR)	16.3 (13.5, 19.5)	7.8 (4.5, 15.3)	14.8 (8.1, 29.7)	15.3 (11.0, 23.0)	12.0 (8.4, 15.4)	10.3 (6.9, 18.9)
Range	(8.6-23.3)	(4.2-19.2)	(6.5-75.1)	(4.7-39.5)	(2.8-44.5)	(4.9-68.0)
Min						
Mean (SD)	151.0 (57.2)	62.4 (11.9)	68.0 (38.2)	134.7 (24.2)	70.8 (16.4)	89.5 (22.5)
Median (IQR)	148 (143, 194)	70 (52, 71)	81 (38, 98)	138 (120, 148)	69 (57, 82)	88 (81, 104)
Range	(62.0-208.0)	(47.0-72.0)	(18.0-105.0)	(62.0-173.0)	(41.0-106.0)	(18.0-125.5)
Max						
Mean (SD)	190.6 (41.7)	89.0 (24.4)	147.0 (64.0)	184.0 (31.5)	107.8 (23.5)	132.3 (31.1)
Median (IQR)	204 (185, 211)	82 (79, 112)	126 (111, 137)	182 (157, 204)	108 (94, 119)	129 (110, 144)
Range	(122.0-231.0)	(57.0-115.0)	(102.0-259.0)	(123.0-246.0)	(57.0-167.0)	(95.0-259.0)
ARV						
Mean (SD)	34.5 (35.7)	9.1 (7.2)	28.7 (32.4)	17.2 (10.5)	12.9 (7.8)	14.3 (12.2)
Median (IQR)	20.1 (19.5, 28.3)	9.1 (2.5, 14.0)	18.2 (3.8, 37.0)	14.7 (10.4, 21.3)	11.9 (8.0, 15.5)	11.7 (7.7, 15.3)
Range	(7.8-97.0)	(1.8-18.3)	(3.5-81.2)	(3.2-49.2)	(2.2-44.6)	(2.4-65.3)
SV						
Mean (SD)	42.2 (33.1)	12.0 (10.4)	37.6 (45.9)	21.8 (13.7)	16.8 (9.1)	18.9 (18.2)
Median (IQR)	36.8 (23.7, 43.0)	10.7 (3.5, 14.0)	25.3 (5.4, 37.0)	18.5 (14.0, 27.1)	16.6 (10.6, 19.1)	15.4 (9.7, 20.9)
Range	(10.3-97.0)	(3.2-28.7)	(4.3-115.8)	(3.7-68.7)	(2.5-49.0)	(3.1-103.2)
CV						
Mean (SD)	11.4 (7.7)	13.8 (8.9)	26.6 (29.8)	10.9 (5.6)	15.0 (7.9)	13.6 (12.7)
Median (IQR)	8.2 (7.4, 13.5)	10.2 (8.3, 16.6)	12.9 (9.1, 27.0)	9.8 (7.0, 13.1)	14.2 (9.6, 18.0)	9.9 (6.8, 15.4)
Range	(4.3-23.8)	(6.0-28.1)	(6.2-78.0)	(2.9-25.2)	(4.3-42.2)	(4.3-71.1)

**Table 3:** Blood pressure variability within 24 hours of clevidipine SD: standard deviation; IQR: interquartile range; ARV: average real variability; SV: successive variation; CV: coefficient of variation; SBP: systolic blood pressure; DBP: diastolic blood pressure; MAP: mean arterial pressure Data is presented as mean (SD) or median (IQR)

	Arterial measures within 24 hours of clevidipine start			All measures within 24 hours of clevidipine start		
	SBP (N = 13)	DBP (N = 13)	MAP (N = 13)	SBP (N = 30)	DBP (N = 30)	MAP (N = 30)
Mean						
Mean (SD)	154.6 (21.1)	75.9 (23.0)	99.5 (19.0)	146.7 (14.6)	76.9 (12.2)	98.3 (10.1)
Median (IQR)	149.1 (140.6, 172.0)	73.7 (68.8, 82.2)	102.2 (88.8, 108.9)	146.6 (135.7, 154.4)	77.0 (70.6, 84.5)	97.6 (89.9, 104.9)
Range	(129.4-200.7)	(45.4-133.4)	(70.9-137.5)	(125.1-180.4)	(46.7-99.2)	(78.8-118.4)
SD						
Mean (SD)	18.4 (7.3)	12.4 (8.5)	17.6 (9.2)	16.4 (5.6)	13.2 (6.0)	14.2 (6.8)
Median (IQR)	18.1 (12.1, 24.8)	11.6 (6.5, 17.0)	14.2 (10.5, 25.9)	16.7 (11.6, 19.7)	12.6 (9.1, 15.9)	11.9 (9.1, 19.0)
Range	(9.4-32.1)	(3.3-34.1)	(5.0-30.6)	(8.2-27.6)	(3.3-35.3)	(5.0-30.1)
Min						
Mean (SD)	109.2 (29.7)	52.8 (13.1)	50.8 (36.4)	102.2 (16.7)	50.3 (12.3)	59.9 (24.7)
Median (IQR)	107 (98, 114)	57 (46, 61)	58 (15, 74)	106 (92, 112)	52 (42, 59)	70 (57, 75)
Range	(62-188)	(27-71)	(0-108)	(62-131)	(22-70)	(0-88)
Max						
Mean (SD)	205.7 (33.0)	127.2 (62.9)	168.5 (76.9)	197.1 (33.4)	135.6 (44.3)	158.5 (54.0)
Median (IQR)	206 (176, 227)	120 (88, 132)	153 (108, 193)	194 (168, 221)	132 (106, 150)	151 (122, 162)
Range	(163-275)	(57-272)	(80-320)	(145-275)	(61-272)	(101-320)
ARV						
Mean (SD)	9.9 (3.6)	6.2 (3.5)	8.0 (4.0)	10.2 (2.9)	8.6 (3.8)	8.3 (3.1)
Median (IQR)	9.5 (7.1, 11.9)	5.9 (3.7, 7.2)	6.5 (5.4, 10.5)	10.6 (7.4, 12.4)	8.2 (6.1, 9.8)	7.8 (6.3, 9.9)
Range	(5.0-16.1)	(2.1-14.5)	(3.4-15.4)	(5.2-15.6)	(2.2-20.9)	(3.5-16.2)
SV						
Mean (SD)	15.3 (5.7)	11.3 (6.8)	15.9 (10.4)	14.5 (4.3)	13.5 (5.9)	14.0 (7.4)
Median (IQR)	15.1 (11.8, 21.3)	9.3 (6.2, 16.3)	10.9 (10.2, 20.5)	15.1 (10.7, 18.4)	13.3 (9.0, 16.5)	11.4 (9.1, 16.0)
Range	(7.0-23.8)	(3.0-22.9)	(4.8-38.0)	(7.4-21.6)	(3.4-31.3)	(4.8-36.4)
CV						
Mean (SD)	12.0 (4.6)	15.3 (6.9)	17.4 (7.6)	11.1 (3.3)	17.0 (7.1)	14.2 (6.2)
Median (IQR)	11.0 (9.1, 14.7)	14.7 (9.1, 21.8)	16.8 (12.4, 22.3)	11.4 (8.6, 13.2)	15.0 (12.9, 20.5)	12.5 (9.8, 17.4)
Range	(5.2-21.5)	(6.4-25.6)	(5.2-29.3)	(5.7-17.9)	(7.0-44.1)	(6.0-28.6)

**Table 4:** Blood pressure variability in intracranial hemorrhage patients ARV: average real variability; SD: standard deviation; SBP: systolic blood pressure; DBP: diastolic blood pressure; MAP: mean arterial pressure Data is presented as mean (SD)

Intracranial hemorrhage patients			
	SBP (N = 19)	DBP (N = 19)	MAP (N = 19)
ARV: 24 hours, mean (SD)	10.8 (3.0)	8.0 (2.9)	8.4 (2.8)
ARV: 1 hour, mean (SD)	17.5 (9.6)	11.4 (5.0)	14.8 (13.3)

Six patients (20%) experienced AKI, and seven (23%) experienced early neurologic decline. The median mRS was 3 (2, 5) at discharge and 4 (1,6) at three months after discharge (Table 5). There was an association between more SBP variability and early neurologic decline as measured by SD, ARV, SV, and CV. Additionally, as measured by CV of diastolic blood pressure (DBP) and mean arterial pressure (MAP), variability in the first hour was associated with neurologic decline (Table 6). BPV did not have an impact on AKI in our cohort (Table 7). Greater variability in SBP and DBP was associated with longer time to blood pressure goal (Table 8). 

**Table 5:** Secondary outcomes IQR: interquartile range Data is presented as N (%) or median (IQR)

	N = 30
Acute kidney injury, n (%)	6 (20)
Neurologic decline, n (%)	7 (23.3)
Time to blood pressure target (minutes)	-
Mean	20.2 (13.8)
Median	20 (10, 28)
Range	(0-57)
Discharge Modified Rankin Scale (mRS) (median IQR)	3 (2, 5)
3-month Modified Rankin Scale (mRS) (median IQR)	4 (1,6)

**Table 6:** Association between blood pressure variability and early neurologic decline ARV: average real variability; SD: standard deviation; SBP: systolic blood pressure; DBP: diastolic blood pressure; MAP: mean arterial pressure; IQR: Interquartile range; ARV: average real variability; SV: successive variation; CV: coefficient of variation * Chi-square test statistic from the Wilcoxon rank sum test. p < 0.05 was considered statistically significant

	SBP				DBP				MAP			
	No neurologic decline (N = 23)	Neurologic decline (N = 7)	Χ^2^*	p-value	No neurologic decline (N = 23)	Neurologic decline (N = 7)	Χ^2^*	p-value	No neurologic decline (N = 23)	Neurologic decline (N = 7)	Χ^2^*	p-value
SD: 24 hours, median (IQR)	16.3 (12.6, 19.2)	18.9 (12.8, 23.0)	0.27	0.61	11.5 (10.5, 15.3)	13.1 (8.6, 20.3)	0.32	0.57	12.3 (9.9, 17.0)	12.3 (10.5, 23.3)	0.07	0.79
ARV: 24 hours, median (IQR)	11.3 (7.4, 12.4)	10.5 (6.9, 12.5)	0.00	0.98	7.8 (6.1, 9.8)	7.5 (5.2, 12.4)	0.03	0.86	7.9 (6.3, 10.4)	7.3 (5.2, 9.8)	0.03	0.86
SV: 24 hours, median (IQR)	14.9 (10.5, 18.4)	17.0 (10.7, 20.4)	0.58	0.45	11.7 (8.9, 16.2)	14.0 (7.5, 17.9)	0.10	0.75	12.0 (9.1, 16.0)	12.2 (10.2, 14.3)	0.03	0.86
CV: 24 hours, median (IQR)	11.2 (8.8, 13.0)	12.9 (8.6, 15.3)	0.14	0.71	15.4 (11.9, 19.2)	14.9 (12.9, 24.6)	0.44	0.51	13.0 (9.8, 16.1)	12.3 (10.0, 21.4)	0.10	0.75
SD: 1 hour, median (IQR)	12.3 (9.7, 17.8)	23.3 (17.0, 36.5)	7.41	0.007	10.0 (7.2, 13.9)	15.3 (9.8, 22.1)	5.66	0.017	8.7 (6.3, 15.3)	19.1 (9.1, 29.7)	3.38	0.066
ARV: 1 hour, median (IQR)	12.6 (7.5, 20.5)	21.0 (14.3, 33.4)	4.34	0.037	11.7 (6.9, 15.4)	15.3 (9.8, 22.1)	2.39	0.12	11.0 (6.6, 14.5)	15.1 (10.8, 19.9)	2.86	0.090
SV: 1 hour, median (IQR)	17.9 (8.5, 26.2)	24.1 (19.4, 42.9)	4.98	0.026	13.5 (9.4, 18.5)	18.1 (13.3, 27.0)	1.56	0.21	13.2 (8.6, 19.5)	20.9 (13.7, 23.8)	3.38	0.066
CV: 1 hour, median (IQR)	8.3 (6.0, 10.5)	13.5 (12.3, 19.0)	8.23	0.004	11.2 (8.3, 16.5)	18.0 (16.6, 25.0)	7.14	0.008	7.2 (6.2, 14.6)	18.4 (8.3, 27.0)	5.20	0.023

**Table 7:** Association between blood pressure variability and AKI AKI: acute kidney injury; SD: standard deviation; IQR: interquartile range; ARV: average real variability; SV: successive variation; CV: coefficient of variation; IQR: interquartile range; SBP: systolic blood pressure; DBP: diastolic blood pressure; MAP: mean arterial pressure * Chi-square test statistic from the Wilcoxon rank sum test A p-value of <0.05 was considered statistically significant

	SBP					DBP					MAP			
	No AKI (N = 24)	Yes AKI (N = 6)	Χ^2^*	p-value	No AKI (N = 24)		Yes AKI (N = 6)	Χ^2^*	p-value	No AKI (N = 24)		Yes AKI (N = 6)	Χ^2^*	p-value
SD: 24 hours, median (IQR)	17.9 (14.3, 20.1)	12.7 (12.0, 13.9)	2.26	0.13	12.6 (9.9, 16.1)		11.2 (10.8, 13.1)	0.69	0.41	12.7 (10.5, 19.5)		11.6 (10.5, 12.3)	1.68	0.19
ARV: 24 hours, median (IQR)	11.5 (8.1, 12.7)	8.9 (5.5, 11.6)	2.26	0.13	7.9 (6.2, 10.7)		6.1 (4.5, 10.5)	1.08	0.30	8.0 (6.4, 10.1)		5.9 (4.3, 8.4)	2.11	0.15
SV: 24 hours, median (IQR)	15.6 (10.9, 19.0)	12.1 (9.2, 14.9)	2.42	0.12	12.5 (9.3, 17.2)		10.8 (7.1, 14.0)	1.08	0.30	11.4 (9.4, 16.7)		12.3 (6.3, 12.5)	0.60	0.44
CV: 24 hours, median (IQR)	12.5 (10.2, 13.4)	8.7 (8.6, 9.2)	2.93	0.087	15.8 (12.8, 20.3)		14.1 (11.9, 15.4)	0.69	0.41	13.3 (10.2, 17.5)		11.2 (9.9, 13.0)	1.68	0.19
SD: 1 hour, median (IQR)	15.3 (11.3, 23.2)	13.8 (11.0, 16.6)	0.17	0.68	12.3 (8.7, 15.9)		10.7 (7.2, 15.4)	0.27	0.60	10.3 (7.1, 17.2)		10.5 (6.4, 19.1)	0.04	0.84
ARV: 1 hour, median (IQR)	14.8 (11.0, 22.7)	11.3 (6.6, 17.9)	1.08	0.30	11.9 (9.0, 15.5)		11.4 (4.2, 19.3)	0.07	0.80	11.7 (8.9, 14.8)		11.0 (6.1, 17.8)	0.10	0.76
SV: 1 hour, median (IQR)	19.1 (14.3, 29.2)	13.4 (7.7, 20.0)	1.55	0.21	16.6 (12.0, 18.8)		13.8 (6.9, 22.3)	0.45	0.50	15.4 (11.3, 20.4)		13.0 (7.1, 21.7)	0.60	0.44
CV: 1 hour, median (IQR)	9.8 (7.4, 13.3)	8.6 (7.0, 11.6)	0.10	0.76	14.8 (10.0, 18.4)		12.0 (9.1, 17.5)	0.33	0.57	9.9 (6.9, 15.3)		9.3 (6.6, 18.4)	0.01	0.92

**Table 8:** Association between blood pressure variability and time to blood pressure goal SD: standard deviation; IQR: interquartile range; ARV: average real variability; SV: successive variation; CV: coefficient of variation; BP: blood pressure; SBP: systolic blood pressure; DBP: diastolic blood pressure; MAP: mean arterial pressure *More variability in SBP/DBP within 1 hour led to longer times to meeting BP goals Results presented are from univariate linear regression models for time to blood pressure goal. A p-value of <0.05 was considered statistically significant

	SBP			DBP			MAP		
	Estimated difference in time to BP goal (95% CI)	t	p-value	Estimated difference in time to BP goal (95% CI)	t	p value	Estimated difference in time to BP goal (95% CI)	t	p-value
SD: 24 hours (per 1 unit increase)	0.21 (-0.84, 1.27)	0.41	0.41	0.51 (-0.38, 1.41)	1.18	0.25	0.13 (-0.72, 0.98)	0.30	0.76
ARV: 24 hours (per 1 unit increase)	0.22 (-1.55, 1.98)	0.25	0.80	0.07 (-1.29, 1.43)	0.11	0.92	-0.42 (-2.15, 1.31)	-0.50	0.62
SV: 24 hours (per 1 unit increase)	0.47 (-0.78, 1.71)	0.77	0.45	0.31 (-0.57, 1.19)	0.72	0.48	-0.20 (-0.99, 0.58)	-0.53	0.60
CV: 24 hours (per 1 unit increase)	-0.01 (-1.76, 1.74)	-0.01	0.99	0.44 (-0.30, 1.19)	1.22	0.23	0.15 (-0.78, 1.08)	0.33	0.74
SD: 1 hour (per 1 unit increase)	0.63 (0.11, 1.16)	2.48	0.020	0.84 (0.25, 1.43)	2.92	0.007	0.14 (-0.28, 0.55)	0.67	0.51
ARV: 1 hour (per 1 unit increase)	0.60 (0.15, 1.05)	2.71	0.011	0.99 (0.42, 1.55)	3.59	0.001	0.16 (-0.28, 0.60)	0.75	0.46
SV: 1 hour (per 1 unit increase)	0.44 (0.09, 0.79)	2.59	0.015	0.82 (0.32, 1.31)	3.39	0.002	0.03 (-0.26, 0.33)	0.22	0.82
CV: 1 hour (per 1 unit increase)	0.54 (-0.39, 1.48)	1.19	0.24	0.74 (0.13, 1.36)	2.47	0.020	0.03 (-0.40, 0.45)	0.12	0.90

## Discussion

To our knowledge, this is the first prospective report on clevidipine safety and efficacy on BVP in acute cerebrovascular disease. This study reports BVP with clevidipine in acute cerebrovascular disease at one hour and 24 hours using six different measures. This information is important because BPV has been associated with unfavorable outcomes in various acute cerebrovascular diseases, but there is a dearth of granular data on BPV in daily practice. Also, this is the first study exploring BPV in patients with acute cerebrovascular diseases being treated with clevidipine infusion.

Randomized controlled trials evaluating acute blood pressure targets in patients with ICH have explored the magnitude of BPV (by hourly blood pressure changes) and its relationship with clinically relevant endpoints. A subgroup analysis from Antihypertensive Treatment of Acute Cerebral Hemorrhage II (ATACH-2) measured BVP via five different measures [13]. In ATACH-2, patients received blood pressure management with nicardipine [14]. BVP was measured in the acute period, defined as 2-24 hours. In comparison to BPV measured in this study with clevidipine, there was less BVP with nicardipine when measured by SD (14.5 ± 5.3 versus 18.4 ± 7.3) and CV (10.3 ± 3.6 versus 12.0 ± 4.6) [13]. However, there was less variability in this study with clevidipine compared to the ATACH-2 subgroup analysis with nicardipine when measured with ARV (9.9 ± 3.6 versus 13.9 ± 6.7) and SV (15.3 ± 5.7 versus 18.0 ± 8.2) [13]. In a preplanned analysis of the Second Intensive Blood Pressure Reduction in Acute Cerebral Haemorrhage Trial (INTERACT-2) and ATACH-2, the SD of systolic BVP for all participants was 14 ± 8, and in the INTERACT-2 group, the SD was 14 ± 8, both showing more variability compared to the SD in our study with clevidipine [15]. In INTERACT-2, the most commonly used blood pressure agent was urapidil, followed by a combined alpha- and beta-blocker [16]. A study by Poyant and colleagues compared BVP in ICH patients who received bolus antihypertensives versus bolus antihypertensives plus continuous infusion of nicardipine [11]. The group who received nicardipine has less BVP compared to the bolus antihypertensive group; however, the specific BVP among the two groups was not reported to compare to the BVP seen in this study.

In a subgroup analysis of the Head Position in Acute Stroke Trial (HEADPOST) study, including both AIS (91% of patients) and ICH, BVP was measured by SD and CV. The SD of the SBP in this study was 11 ± 7.5 mmHg, and the CV was 8 ± 4.4 mmHg within the first 24 hours. However, it was not reported if patients required pharmacologic treatment for blood pressure management and, if so, what agents were administered [1].

Our study found early neurologic decline in 23% of patients. In ICH patients, INTERACT-2 found neurologic decline in approximately 15% of patients, and ATACH-2 found that approximately 10% of patients had neurologic decline. In our ICH patients, 21% (4/19) had neurologic decline. ATACH-2 found renal adverse events in 9% in their intensive treatment group and 4% in their standard treatment group [15]. In our patients, BPV was associated with neurologic decline; however, we were unable to control for other factors that may contribute to neurologic decline. In this study, 20% of patients had AKI, but our definition varied from the ATACH-2 trial, and we were unable to control for other factors in critical illness that contribute to AKI.

Blood pressure goal was rapidly achieved with clevidipine, after a median of 20 minutes (10, 28). Previous studies in acute cerebrovascular disease have shown clevidipine to reach the blood pressure goal between 30 and 65 minutes [17,18]. In comparison, the time to blood pressure goal with nicardipine has been reported between 30 and 74 minutes, and labetalol has reached blood pressure goal between 53 and 90 minutes in cerebrovascular emergencies [17-24]. Thus, clevidipine can rapidly achieve blood pressure goals and should be considered a good option for future trials of acute hypertension treatment in patients with cerebrovascular emergencies. Yet, in order to minimize the potential deleterious consequences of excessive BPV, we think that such future trials should target percentage blood pressure reductions (e.g., 20% from initial SBP) rather than absolute blood pressure values.

Our study is limited by the small number (n = 30), heterogeneity of the acute cerebrovascular emergencies included, and single-arm design. We cannot determine if the direct association between BPV and the chances of early neurological decline was caused by greater BPV or if increased BPV may be a consequence of the neurological decline. Numerous factors contribute to early neurologic decline and AKI that are unable to be controlled for in this study, and these results should be considered exploratory. Additionally, we acknowledge the heterogeneity in BPV measurements, making it difficult to compare our results to previous literature and the numerous measurements utilized. It was conceived as an initial investigation to evaluate the effects of a practice of immediate introduction of a potent and fast-acting vasodilator infusion on measures of BPV and clinical events. These results should serve as a basis for more research comparing BVP and clinical events in patients treated with different antihypertensive protocols.

## Conclusions

Clevidipine may be an effective option to rapidly reach the blood pressure target in patients with cerebrovascular emergencies. BPV was associated with early neurologic decline and longer time to blood pressure control; however, the small sample size and observational study are the limitations of this study. Comparative studies evaluating the effects of different antihypertensive strategies on BPV are needed.
